# Pre-pregnancy Body Mass Index (BMI) and delivery outcomes in a Canadian population

**DOI:** 10.1186/s12884-014-0422-y

**Published:** 2014-12-20

**Authors:** Angela Vinturache, Nadia Moledina, Sheila McDonald, Donna Slater, Suzanne Tough

**Affiliations:** Departments of Paediatrics/Physiology & Pharmacology, Cumming School of Medicine, Alberta Centre for Child, Family & Community Research - Child Development Centre, University of Calgary, c/o 2888 Shaganappi Trail NW, Calgary, AB T3B 6A8 Canada; Faculty of Medicine, University of Alberta, 2J 2.00 WC Mackenzie Health Science Centre, Edmonton, AB T6G 2R7 Canada; Department of Pediatrics, Cumming School of Medicine, Alberta Centre for Child, Family & Community Research - Child Development Centre, University of Calgary, c/o 2888 Shaganappi Trail NW, Calgary, AB T3B 6A8 Canada; Department of Physiology & Pharmacology, Cumming School of Medicine, University of Calgary, # 277 HRMB Building, 3330 Hospital Drive NW, Calgary, AB T2N 4N1 Canada; Departments of Pediatrics and Community Health Sciences, Cumming School of Medicine, Alberta Centre for Child, Family & Community Research, Child Development Centre, University of Calgary, c/o 2888 Shaganappi Trail NW, Calgary, AB T3B 6A8 Canada

**Keywords:** Pregnancy, Obesity, Overweight, Labour induction, Caesarean section, Pregnancy complications

## Abstract

**Background:**

Worldwide there has been a dramatic increase in the prevalence of overweight and obesity in women of childbearing age. Growing evidence suggests that maternal overweight and obesity is associated with poor maternal and perinatal outcomes. This study evaluated the impact of maternal pre-pregnancy overweight and obesity on pregnancy, labour and delivery outcomes in a cohort of women with term, singleton pregnancies cared for by family physicians in community based practices.

**Methods:**

This study is a secondary analysis of the All Our Babies Cohort, a prospective, community-based pregnancy cohort in Calgary, Alberta. Maternal self-reported data on height and pre-pregnancy weight from term, singleton, cephalic pregnancies (n = 1996) were linked to clinical data on pregnancy and birth events retrieved from electronic health records. Descriptive and bivariate regression analysis were used to compare pregnancy and birth outcomes between women categorized as normal weight, overweight and obese based on the pre-pregnancy BMI. Multinomial regression analysis stratified by type of labour onset examined the association between pre-pregnancy BMI and mode of delivery controlling for maternal age, pre-existent health conditions, parity, fertility treatments, history of C-section and pregnancy complications.

**Results:**

The cohort consisted of 65.8% normal weight, 23.6% overweight and 10.6% obese women. Women with increased pre-pregnancy BMI were more likely to develop pregnancy complications such as preeclampsia (OR 3.5, CI 2.0-4.6 for overweight; OR 5.3, CI 3.3-8.5 for obese) and gestational diabetes (OR 3.0, CI 1.8-5.0 for overweight; OR 6.5, CI 3.7-11.2 for obese) than normal weight women. Spontaneous onset of labour was recorded in 71.2% of women with normal pre-pregnancy BMI, whereas 39.3% of overweight and 49% of obese women had their labour induced. For women with spontaneous labour, pre-pregnancy BMI was not a significant risk factor for mode of delivery, controlling for covariates. Among women with induced labor, obesity was a significant risk factor for delivery by C-section (adjusted OR 2.2; CI 1.2-4.1).

**Conclusions:**

Even among women with term, singleton pregnancies obtaining prenatal care in community-based settings, obese women who undergo labour induction are at increased risk of obstetrical interventions at delivery. These findings highlight the importance of tailored maternal care in pregnancy and at delivery of pregnant women with increased BMI in order to improve the outcomes and wellbeing of these women and their children.

**Electronic supplementary material:**

The online version of this article (doi:10.1186/s12884-014-0422-y) contains supplementary material, which is available to authorized users.

## Background

Obesity has emerged as a major public health problem around the globe over the past two decades [1,2]. As the overall prevalence of obesity increases so does the number of women of reproductive age who are overweight or obese. In Canada, the prevalence of obesity among women aged 20 to 39 is 21% [3], and almost one third of women of reproductive age in the USA are reported to be obese [4].

The association between maternal obesity and pregnancy and labour outcomes is complex. Emerging evidence suggests obesity is associated with increased complications during pregnancy, labour and delivery, and into the postpartum period, as well as adverse neonatal outcomes which include fetal growth abnormalities such as macrosomia [5,6], neural tube defects [7], and stillbirth [8,9]. These have implications for obstetrical management and maternal and neonatal care [10-12]. In addition to the deleterious impact on the overall health of pregnancy, obesity may also affect clinical decisions for the management of labour and delivery, which ultimately may have repercussions on health care costs and maternity services [13]. Accumulating evidence suggests that obesity contributes to the increased rates of labour induction [14,15] and obstetrical interventions [16,17]. Labour progression is significantly slower in obese women [18,19] whereas duration of labour, oxytocin requirements and caesarean delivery rates increase with increasing maternal body mass index (BMI) [19].

Importantly, there appears to be a linear relationship between maternal BMI and obstetrical interventions at birth [6]. That is, not only obese, but also overweight women are at increased risk of adverse pregnancy and neonatal outcomes [6]. While current knowledge on the complex interactions between obesity and pregnancy outcomes has increased awareness over the past decade to this modifiable risk factor for maternal and neonatal health, a number of gaps remain. Much of our information on adverse outcomes of obesity on maternal and neonatal health comes from large cohorts drawn retrospectively from birth registries or hospital-based studies, which are primarily based on administrative data. Prospective, community-based studies may offer the advantage of detailed data collection on other factors that may influence outcomes such as marital status, ethnicity, alcohol, drug, tobacco use, fertility treatments and other maternal conditions. In addition, prospective cohorts may provide valuable information on the preponderance of local environmental, demographic and nutritional risk factors associated with obesity in pregnancy and reveal particularities of different populations, thus providing platforms for more targeted and effective interventions to address the health needs of women of childbearing age from a certain geographical area.

Therefore, the aim of this study was to assess the impact of increased BMI prior to pregnancy on maternal complications, delivery and labour outcomes in overweight and obese women from a community-based longitudinal pregnancy cohort from Calgary, Alberta metropolitan area.

## Methods

### Data source

Data for this study was drawn from the All Our Babies (AOB) study, a prospective community-based pregnancy cohort in Calgary, Alberta whose overall objectives were to examine maternal well-being and health service utilization across the perinatal period. Participants were recruited into the AOB between May 2008 and December 2010 and included in the study if they were older than 18 years of age, less than 25 weeks pregnant, and received prenatal care in primary health care offices. Detailed demographic, environmental and lifestyle information have been collected through questionnaires administered during the prenatal period, before 25 weeks, at 34–36 weeks of gestation, and at 4 months postpartum. Information on recruitment, eligibility, data collection and questionnaires utilized in the AOB study is described in detail elsewhere [20,21]. The questionnaires were pilot tested and included both standardized tools when available or questions specifically created with input from health care providers, community care programs experts and epidemiologists when standardized items were not suitable [20].

The self-reported information on women’s experiences during pregnancy, birth outcomes and post-partum period obtained from the surveys were linked to the electronic health records at hospital admission for labour and delivery according to unique personal health care numbers. The medical records provided additional and pertinent details on pregnancy complications and birth outcomes not captured on the surveys. Ethical approval for this study was obtained from the Conjoint Health Research Ethics Board of the University of Calgary. Written informed consent was obtained from the study participants at the time of recruitment, who were also provided copies for their records.

### BMI calculation and grouping

Information on weight and height prior to pregnancy was collected at the first data collection time point (<25 weeks) via maternal report. Pre-pregnancy BMI was calculated as the ratio of weight prior to pregnancy (kg) divided by height (m^2^). Women were divided into five groups based on pre-pregnancy BMI according to categories defined by World Health Organization’s classification [22] and Health Canada Guidelines recommendation [23] as follows: underweight (BMI < 18.50 kg/m^2^), normal weight (BMI 18.50-24.99 kg/m^2^), overweight (BMI 25.00-29.99 kg/m^2^), obese (BMI 30.00-39.99 kg/m^2^), and morbidly obese (BMI > 40.00 kg/m^2^).

Given the small number of women meeting the criteria for “morbidly obese” (n = 31), for meaningful comparisons this category was combined with the “obese” category and collectively referred as obese. Statistical comparisons were carried out between three BMI groups: (1) “normal weight”, (2) “overweight”, and (3) “obese”.

### Study sample

The inclusion criteria were: 1) participation in the AOB study and completion of all three questionnaires; 2) the survey data could be linked to the medical records; 3) pre-pregnancy BMI ≥ 18.5 kg/m^2^; 4) clinical characteristics: singleton pregnancies, cephalic presentation, delivery at term (≥37 weeks gestation). Exclusion criteria were: 1) missing data (i.e. height or pre-pregnancy weight not recorded) (n = 457); 2) pre-pregnancy BMI < 18.5 kg/m^2^ (n = 149); 3) clinical characteristics: multiples (n = 36), planned caesarean section (n = 364) or preterm delivery (<37 weeks gestation) (n = 208). From 3388 women recruited in the AOB, 1996 met the criteria above and were included in the present study.

### Outcome measures

The outcomes of labour and delivery were compared between normal weight, overweight and obese women. The main outcome measures included type of labour onset (spontaneous labour, induced labour) and mode of delivery (operative vaginal, caesarean deliveries, spontaneous vaginal deliveries). In additional analyses, pre-pregnancy BMI and the elevated risk of pregnancy complications pregnancy was described.

In order to determine the independent relationship between mode of delivery and pre-pregnancy BMI we controlled for maternal age, obstetrical history (parity), fertility treatments (patients who achieved the pregnancy by artificial or partially artificial means, i.e. underwent artificial reproductive techniques or medical treatments such as induction of ovulation), and pre-existent health conditions (i.e. hypertension, diabetes, chronic renal and cardiac diseases). Pregnancy complications (pregnancy-induced hypertension, preeclampsia, eclampsia, placental abruption, gestational diabetes, prolonged rupture of membranes, placenta praevia) were also considered covariates, recognizing that controlling for these potentially intermediary conditions would provide us with a conservative estimate of the influence of BMI on outcomes.

### Statistical analysis

Descriptive statistics were produced for all study variables. Continuous variables were presented as the mean and standard deviation (SD) or mean (95% confidence intervals). Categorical data were presented as the frequency and percentage. Gestational age and birth weight were examined as both continuous and categorical variables.

Bivariate analysis was performed to examine the associations between pre-pregnancy BMI categories and maternal demographics, lifestyle characteristics and pregnancy complications, using Chi square test for categorical variables and ANOVA for continuous variables, as appropriate.

Multivariable logistic regression analysis was used to examine the association between pre-pregnancy BMI and type of labour onset, controlling for maternal age, parity, pre-existing maternal conditions, fertility treatments, and pregnancy complications.

Because mode of delivery had three categories (operative vaginal, C-section, spontaneous vaginal), multinomial logistic regression models were constructed to examine the association between maternal pre-pregnancy BMI (normal weight, overweight and obese), and mode of delivery, controlling for maternal age, parity, pre-existing health conditions, fertility treatments, history of C-section and pregnancy complications. This analysis was further stratified by type of labour onset (spontaneous vs. induced). Women who delivered by spontaneous vaginal delivery were the reference group. A stepwise model building strategy was adopted for the regression analyses, with non-modifiable (e.g. demographics) and previously identified obstetrical and medical factors entered in sequential blocks.

Comparisons between the pre-pregnancy BMI groups were made using the normal weight group of women as the reference category. Where appropriate unadjusted (OR) and adjusted odds ratio (aOR) and their 95% confidence intervals (CI) were computed; a value of p < 0.05 was considered statistically significant. All statistical analyses were performed using the SPSS for Windows package, versions 20 (IBM SPSS, Chicago, IL).

## Results

### Baseline characteristics

The socio-demographic characteristics of the study sample were similar to the characteristics describing the AOB cohort [20] (Additional file 1: Table S1). The mean maternal age of participants was 30.6 (4.4) years, range 18–43 in our study compared to 31.0 (4.5) years, range 18–47 (only 3 participants older than 43 years) in the parent study, AOB [20].

The majority of the sample was Caucasian (80.4%), younger than 35 years of age (78.3%), had a partner (95.4%), had completed postsecondary education (90.5%) and reported an annual household income of at least $80,000 Canadian dollars (72.5%) (Table 1). These characteristics of our sample mirror the AOB cohort characteristics and align with the pregnant and parenting population of an urban centre in Canada, with the exception of higher incomes [20].

**Table 1:** **Baseline socio-demographic and lifestyle characteristics of the participants in the study according to their pre-pregnancy body mass index (BMI)**

**Characteristic**	**Full sample (N)** ^**1**^	**Normal weight (BMI 18.5-24.9 kg/m** ^**2**^ **)**	**Overweight (BMI 25–29.9 kg/m** ^**2**^ **)**	p*	**Obese** **(BMI ≥ 30 kg/m** ^**2**^ **)**	***p*** ^***†***^
**N (%)**	1996 (100%)	1313 (65.8%)	472 (23.6%)		211 (10.6)	
**Demographics**						
Maternal age				0.279		0.261
≤34 years old	1539 (78.3)	1011 (78.5)	356 (76.1)		172 (81.9)	
≥35 years old	427 (21.7)	277 (21.5)	112 (23.9)		38 (18.1)	
Ethnicity				**0.025**		**0.005**
Caucasian	1602 (80.4)	1027 (78.4)	392 (83.2)		183 (86.7)	
Other	390 (19.6)	283 (21.6)	79 (16.8)		28 (13.3)	
Household income				0.451		**0.035**
≤ $39,000	139 (7.2)	87 (6.8)	34 (7.4)		18 (8.7)	
$40,000- $79,999	394 (20.3)	243 (19.0)	98 (21.4)		53 (25.7)	
≥ $80,000	1406 (72.5)	946 (74.1)	325 (71.1)		135 (65.5)	
Education				**<0.001**		**<0.001**
High school	190 (9.5)	108 (8.3)	47 (10.0)		35 (16.6)	
Post-secondary	1802 (90.5)	1201 (91.7)	425 (90.0)		176 (83.4)	
Marital status				0.961		0.450
Single	92 (4.6)	59 (4.5)	21 (4.4)		12 (5.6)	
Married/common law	1901 (95.4)	1251 (95.5)	451 (95.6)		199 (94.3)	
**Lifestyle**						
Smoking during pregnancy				0.416		**0.033**
No	1894 (94.9)	1248 (95.0)	453 (96.0)		193 (91.5)	
Yes	102 (5.1)	65 (5.0)	19 (4.0)		18 (8.5)	
Alcohol use in pregnancy^2^				**0.018**		0.288
No	1981 (99.2)	1306 (99.5)	464 (98.3)		211 (100.0)	
Yes	15 (0.8)	7 (0.5)	8 (1.7)		0 (0.0)	
Recreational drugs use in pregnancy				0.285		0.571
No	1992 (99.8)	1311 (99.8)	470 (99.6)		211 (100.0)	
Yes	4 (0.2)	2 (0.2)	2 (0.4)		0 (0.0)	

Data presented as n (%) (χ^2^ test) for categorical variables or mean (95% confidence intervals) (analysis of variance ANOVA) for continuous variables.

*Comparison between normal weight and overweight women.

^†^Comparison between normal weight and obese women.

^1^May not add to total number (N = 1996) due to missing.

^2^Refers to consumption of ≥3 drinks on any one occasion at any time during pregnancy.

Omnibus test p-values were significant for ethnicity (p = 0.004), education (p = 0.001), smoking (p = 0.043), and alcohol use in pregnancy (p = 0.018) and not significant for any of the other variables included in the table.

Percentages are calculated per column for each variable.

BMI, body mass index.

Comparisons between women from the three BMI categories by demographics, obstetrical and lifestyle factors are shown in Table 1. Among 1996 women included in the study, 1313 (65.8%) were of normal pre-pregnancy weight, 472 (23.6%) were overweight and 211 (10.6%) were obese or morbidly obese. Overweight and obese women were more likely to be Caucasian and attain lower levels of education. In addition, a higher proportion of obese women had yearly household incomes less than 80,000/year and smoked during pregnancy.

### Obstetrical characteristics

The obstetrical characteristics of the participants are summarized in Table 2. There were no differences in our sample between normal weight women and women with increased BMI regarding the number of previous pregnancies and births, mode of conception or gestational age at delivery. The higher the maternal BMI before conception, the lower the likelihood of spontaneous onset of labour at term. Spontaneous onset of labour was recorded in 71.2% of women with normal pre-pregnancy BMI compared to 60.7% of overweight and 51% of obese women (p < 0.001). Conversely, labour induction was more frequent in obese women; almost half (49%) of the obese women from our sample had their labour induced. However, no differences were observed in the induction methods used between the groups, with oxytocin being the preferred method used, followed by amniotomy and other methods (i.e. prostaglandins). Regarding mode of delivery, the majority of the sample (77.3%) had a spontaneous vaginal birth. From the 22.7% women who delivered by an obstetrical intervention, 12.7% delivered by emergency C-section and 10% by assisted vaginal delivery, forceps and/or vacuum. The highest rate of emergency C-section was observed in the obese women (21.1%), who also had the lowest rate of operative vaginal deliveries (3.9%). In normal weight women, the C-section rate was significantly lower, 11.2%. There was no difference in the duration of the second and third stage of labour for both operative and spontaneous vaginal deliveries between women in the three BMI categories.

**Table 2:** **Obstetric characteristics of the participants in the study according to their pre-pregnancy body mass index (BMI)**

**Obstetrics characteristic**	**Total sample (N = 1996)** ^**1**^	**Normal weight (BMI 18.5-24.9 kg/m** ^**2**^ **)**	**Overweight (BMI 25–29.9 kg/m** ^**2**^ **)**	p*	**Obese (BMI ≥ 30 kg/m** ^**2**^ **)**	***p*** ^***†***^
Gravidity				0.488		0.142
Primigravida	774 (38.9)	522 (39.9)	179 (38.1)		73 (34.6)	
Multigravida	1215 (61.1)	786 (60.1)	291 (61.9)		138 (65.4)	
Parity				0.416		0.348
Primiparous	1045 (52.4)	699 (52.3)	241 (51.1)		105 (49.8)	
Multiparous	951 (47.6)	614 (46.8)	231 (48.9)		106 (50.2)	
Assisted conception^2^				0.125		0.395
No	1864 (97.1)	1234 (94.3)	435 (92.4)		195 (92.9)	
Yes	58 (2.9)	74 (5.7)	36 (7.6)		15 (7.1)	
Gestational age at delivery				0.744		0.422
37^0/7^ – 41^6/7^ weeks (term)	1991 (99.7)	1309 (99.7)	471 (99.8)		211 (100)	
≥ 42^0/7^ weeks (post-term)	5 (0.3)	4 (0.3)	1 (0.2)		0 (0.0)	
Type of labour				**<0.001**		**<0.001**
Spontaneous	1285 (66.6)	904 (71.2)	277 (60.7)		104 (51.0)	
Induced	644 (33.4)	365 (28.8)	179 (39.3)		100 (49.0)	
Method of induction^3^						
Oxytocin				0.100		0.197
No	138 (21.4)	88 (24.1)	32 (17.9)		18 (18.0)	
Yes	506 (78.6)	277 (75.9)	147 (82.1)		82 (82.0)	
Amniotomy				0.630		0.596
No	392 (60.9)	226 (61.9)	107 (59.8)		59 (59.0)	
Yes	252 (39.1)	139 (38.1)	72 (40.2)		41 (41.0)	
Other methods^4^				0.485		0.605
No	504 (78.3)	287 (78.6)	136 (76.0)		81 (81.0)	
Yes	140 (21.7)	78 (21.4)	43 (24.0)		19 (19.0)	
Mode of delivery				0.236		**<0.001**
Spontaneous vaginal delivery	1492 (77.3)	998 (78.6)	341 (74.8)		153 (75.0)	
Operative vaginal delivery	192 (10.0)	129 (10.2)	55 (12.1)		8 (3.9)	
Caesarean section	245 (12.7)	142 (11.2)	60 (13.2)		43 (21.1)	
Labour duration in spontaneous vaginal deliveries						
Second stage of labour (min)	64.4 (68.6)	66.1 (61.7-70.5)	62.6 (55.8-69.3)	1.000	57.7 (47.6-67.8)	0.448
Third stage of labour (min)	7.7 (9.2)	7.81 (7.2-8.4)	7.6 (6.8-8.4)	1.000	7.1 (5.8-8.3)	1.000
Labour duration in operative vaginal deliveries						
Second stage of labour (min)	115.8 (87.7)	116.6 (100.4-132.7)	108.2 (88.2-128.2)	1.000	157.3 (88.7-225.7)	0.614
Third stage of labour (min)	6.1 (6.5)	6.35 (5.1-7.6)	5.89 (4.7-7.1)	1.000	4.0 (2.7-5.2)	0.983
Obstetrical analgesia (epidural)				**0.017**		**<0.001**
No	752 (39.0)	532 (41.9)	162 (35.5)		58 (28.4)	
Yes	1177 (61.0)	737 (58.1)	294 (64.5)		146 (71.6)	

Data presented as n (%) (χ^2^ test) for categorical variables or mean (95% confidence intervals) (analysis of variance ANOVA) for continuous variables.

^1^May not add to total number (N = 1996) due to missing.

^2^Refers to using fertility treatments to achieving the pregnancy such as fertility enhancing drugs, artificial insemination and artificial reproductive techniques (*in vitro* fertilization, intracytoplasmic sperm injection, fresh and donor embrio transfer etc.).

^3^Other interventions including prostaglandins for induction of labour as classified by Canadian Classification of Health Interventions under section 5, antepartum interventions.

*Comparison between normal weight and overweight women.

^†^Comparison between normal weight and obese women.

Omnibus test p-values were found significant for type of labour onset (p < 0.001), mode of delivery (p < 0.001), and epidural analgesia (p < 0.001) and not significant for any other variables included in the table.

Percentages are calculated per column for each variable.

BMI, body mass index.

Because the rates of labour induction and C-section were higher in women with increased BMI, we further examined the association between the type of labour onset, induced versus spontaneous, and the rates of C-section in obese and overweight women.

### Primary outcomes

In multivariable analysis, the risk for labour induction was increased among women who were overweight (aOR 1.8, CI 1.3-2.5) and obese (aOR 1.3, CI 1.0-1.7) and among women with pregnancy complications (aOR 7.3, CI 5.6-9.7). Multiparity decreased the probability of labour induction (aOR 0.5, CI 0.4-0.7). Maternal age and general health, mode of conception and history of C-section did not influence the probability of induced onset of labour (Table 3).

**Table 3:** **Multivariable logistic regression for labour induction**

**Risk factors for labour induction** ^**1**^	**OR; 95% CI**	**aOR; 95% CI**
Maternal age		
≥35 years old	1.1; 0.9-1.4	1.2; 0.9-1.5
<35 years old	1.0	1.0
Pre-existent medical conditions^2^		
Yes	1.4; (1.1-1.9)*	1.1; 0.8-1.5
No	1.0	1.0
Parity		
Multiparity	0.5; (0.4-0.6)**	0.5; 0.4-0.7**
Primiparity	1.0	1.0
Pregnancy complications^3^		
Yes	8.3; (6.4-10.9)**	7.3; 5.6-9.7**
No	1.0	1.0
Fertility treatments^4^		
Yes	2.0; (1.4-2.9)**	1.4; 0.9-2.2
No	**1.0**	1.0
Pre-pregnancy BMI		
Overweight	1.6; (1.2-2.0)**	1.3; 1.0-1.7*
Obese	2.3; (1.7-3.2)**	1.8; 1.3-2.5*
Normal weight	1.0	1.0

*Abbreviations*: *OR* unadjusted odd s ratio, *aOR* adjusted odds ratio, *CI* confidence interval, *C-section* caesarean section, *BMI* body mass index.

^1^Reference category: spontaneous labour.

^2^Pre-existing diabetes mellitus, hypertension, chronic heart disease, chronic renal diseases.

^3^Gestational diabetes, preeclampsia, eclampsia, placental abruption, placenta praevia, prolonged rupture of membrane, IUGR.

^4^Includes achieving the pregnancy through fertility enhancing drugs, artificial insemination and artificial reproductive techniques (*in vitro* fertilization, intracytoplasmic sperm injection, fresh and donor embrio transfer etc.).

*p < 0.05; **p < 0.001.

When controlling for other factors such as maternal age and pre-existent health conditions, parity, mode of conception, pregnancy complications and history of C-section, multinomial regression models showed that overweight women were not at increased risk for emergency C-section or operative vaginal delivery, regardless of the type of labour onset, spontaneous or induced (Table 4). However, obese women were twice more likely (aOR 2.2, CI 1.2-4.1) to deliver by emergency C-section if their labour was induced, controlling for the same factors. These analyses also showed that parity was independently associated with the mode of delivery, being a protective factor for delivery by obstetrical intervention (both C-section and vacuum and/or forceps) if the labour was either spontaneous or induced (Additional file 2: Table S2). As expected, obstetrical surgical history was an independent risk factor for mode of delivery, previous delivery by C-section significantly increasing the risk of delivery by emergency C-section, regardless of the type of labour onset.

**Table 4:** **Multinomial regression model for the association between mode of delivery and maternal pre-pregnancy BMI by stratified by type of labour onset**

**Mode of delivery**	**Overweight** ^**1**^ **(BMI 25–29.9 kg/m** ^**2**^ **)**		**Obese** ^**1**^ **(BMI ≥ 30 kg/m** ^**2**^ **)**	
	***Spontaneous labour***	***Induced labour***	***Spontaneous labour***	***Induced labour***
Operative vaginal delivery	1.1; 0.7-1.8	1.5; 0.8-2.7	0.3; 0.1-1.0	0.4; 0.1-1.4
Caesarean section	1.1; 0.6-1.8	1.2; 0.7-2.0	1.5; 0.7-3.0	2.2; 1.2-4.1*
Spontaneous vaginal delivery	1.00	1.00	1.00	1.00

Data presented as adjusted odds ratio; 95% confidence intervals.

Adjusted for: maternal age, parity, pre-existent health conditions (diabetes mellitus, hypertension, chronic heart disease, chronic renal diseases), pregnancy complications (gestational diabetes, preeclampsia, eclampsia, placental abruption, placenta praevia, prolonged rupture of membrane, IUGR), fertility treatments, previous C-section.

^1^Reference category: normal weight, BMI 18.5-24.9 kg/m^2^.

*p **=** 0.011.

### Secondary outcomes

Table 5 illustrates increased frequency of adverse pregnancy outcomes in overweight and obese women compared to women with normal pre-pregnancy BMI. Women who were overweight and obese were more likely to develop pregnancy complications such as pregnancy-induced hypertension, preeclampsia, and gestational diabetes. The risk of these complications increased with increasing BMI. However, women who were overweight had elevated risk for bleeding during pregnancy and amniotic fluid disorders, whereas eclampsia was more likely to occur in obese women. No differences were observed in the rates of intrauterine growth restriction, chorioamnionitis, and intra-partum fever between normal weight, overweight and obese women.

**Table 5:** **Relationship between obstetric complications during pregnancy and pre-pregnancy body weight (n = 1996)**

	**Pre-pregnancy BMI categories**				
**Obstetrics complications**	**Normal weight (BMI 18.5-24.9 kg/m** ^**2**^ **)**	**Overweight (BMI 25–29.9 kg/m** ^**2**^ **)**	**Unadjusted OR; 95% CI** ^**2**^	**Obese (BMI ≥ 30 kg/m** ^**2**^ **)**	**Unadjusted OR; 95% CI** ^**3**^
Pregnancy induced hypertension	46 (3.5)	49 (10.4)	3.1; 2.1-4.6**	33 (15.6)	5.7; 3.7-8.8**
Preeclampsia	47 (3.6)	48 (10.2)	3.5; 2.0-4.6**	35 (16.6)	5.3; 3.3-8.5**
Abruptio placentae	16 (1.2)	4 (0.8)	1.4; 0.5-4.3	1 (0.5)	2.6; 0.3-19.6
Eclampsia	3 (0.2)	3 (0.6)	2.8; 0.5-13.8	5 (2.4)	10.6; 2.5-44.6**
Gestational diabetes mellitus	28 (2.1)	27 (5.7)	3.0; 1.8-5.0**	23 (10.9)	6.5; 3.7-11.2**
Intrauterine growth restriction	32 (2.4)	7 (1.5)	1.6; 0.7-3.8	1 (0.5)	5.3; 0.7-38.6
Maternal pyrexia during labour	57 (4.3)	18 (3.8)	1.1; 0.7-1.9	13 (6.2)	0.7; 0.4-1.3
Chorioamnionitis	19 (1.4)	7 (1.5)	1.1; 0.4-2.8	4 (1.9)	0.7; 0.3-2.3
Bleeding during pregnancy	78 (5.9)	47 (10.0)	1.7; 1.2-2.5*	14 (6.6)	1.1; 0.6-2.0
Amniotic fluid disorders^1^	29 (2.2)	20 (4.2)	1.9; 1.1-3.5*	7 (3.3)	1.5; 0.6-3.5

Data presented as n (%).

^1^Includes both polyhydramnions and oligohydramnios.

^2^Unadjusted odds ratios refers to comparisons between normal weight and overweight.

^3^Unadjusted odds ratios refers to comparisons between normal weight and obese.

*p < 0.05; **p < 0.001.

## Discussion

In this study, we report that obesity affects 10% of women of childbearing age in a Canadian urban setting, which is similar to Nova Scotia’s Atlee perinatal database [24] but slightly higher than international assessments of obesity in pregnant women of 8% in Spain [25] and 6% in Australia [26]. These differences may reflect differences in social and dietary habits between countries and continents as well as the global trend of increasing in prevalence of obesity in general population. Evidence across different obstetric populations is consistent that increased pre-pregnancy BMI associates with increased perinatal morbidity, including obstetrical interventions at birth such as labour induction and surgical deliveries [13,24,27,28]. In support of these reports, our study showed that the likelihood of labour induction increased with increased pre-pregnancy BMI, and that obese women were more likely to deliver by C-section. Barau *et al.* also found a linear trend between pre-pregnancy BMI and the rates of caesarean section, with an OR of 1.89 for normal weight, 2.31 for overweight and 2.71 for obese women, however, they included in their analysis elective caesareans and did not control for prior caesareans and induction [29]. After controlling for parity and prior C-section, Kominiarek *et al.* found the relative risk of delivery by C-section to be three times higher in nulliparas and multiparas with BMI ≥ 40 kg/m^2^ compared with the reference group with BMI < 25 kg/m^2^ [30]. Other studies have shown a proportional increase in the risk of caesarian delivery corresponding to the level of maternal obesity [6,19,27,29-32], that was largely attributed to the increased likelihood of pregnancy-related complications in obese women, such as preeclampsia, diabetes, fetal macrosomia and consequent labour inductions.

However, studies to date have not stratified the delivery outcome by the type of labour onset. In this study, we showed that obese women who were induced were more likely to deliver by C-section. Additionally, among women with spontaneous onset of labour no differences were apparent in duration of second and third stage of labour and obstetrical interventions at birth between overweight or obese and women with normal body weight prior to conception. This suggests that although obesity in pregnancy is not an independent justification for labour induction [33], obese women are more likely to be induced and if induced are more likely to undergo delivery by C-section.

The twofold increase in the risk of C-section rates in obese women after induction was independent of pregnancy complications, parity, prior caesarean deliveries, chronic maternal health conditions, treatments for infertility, or maternal age. Thus, other factors may have contributed to our findings. One hypothesis for the increased risk of C-section subsequent to induction includes altered uterine contractility combined with dysfunctional labour which may increase the rate of emergent surgical interventions [34,35]. Furthermore, priming the myometrium for transitioning from quiescence to contractility may be altered with increased BMI and adipose tissue mass [35]. The present findings point to such possible mechanisms in obese but not in overweight women. In addition, the alterations in function appear to occur under conditions of labour induction when the transition from uterine quiescence to active contractility is introduced mechanistically and does not occur at physiologic pace. To date, no BMI thresholds have been reported above which the rates of labour dystocia, and consequently operative delivery, climbs significantly. Future research focused on understanding labor mechanisms may provide insights into the molecular mechanisms that govern myometrial contractility and explain a potential causal relationship between obesity and increased risks at birth.

The association between higher pre-pregnancy BMI and increased risk of C-section delivery in women with induction of labour is of clinical and public health importance. If the trend towards increased pre-pregnancy BMI persists and these women remain at elevated risk of labour induction, then the C-section rate would be expected to increase. Our findings demonstrate that increased pre-pregnancy BMI adversely influence pregnancy outcomes and obstetrical management at birth even among women receiving obstetrical care in community based settings. This study alludes to the missed opportunities in the routine care to address the issue of obesity prior to pregnancy, and advocates for the importance of preconception counseling and weight management prior to pregnancy for optimal pregnancy and birth outcomes.

This study is limited by reliance on self-reported pre-pregnancy BMI data, which may have led to under reporting [36,37]. Under reporting of BMI would render these findings more conservative and as such, the risks associated with BMI may be underestimates of the true risk [38]. Previous publications from our group demonstrate a high level of agreement between maternal self-report on demographics, environmental, and obstetrical information and the corresponding data from the electronic medical records [39]. Another limitation of the current study includes collapsing obese and extremely obese groups into a single group. As the size of our severely obese group was relatively small, we could not perform further subgroup analyses of all BMI categories as defined by the Institute of Medicine and World Health Organization [1,2]. Finally, it is possible that controlling for pregnancy complications in an effort to determine the independent effect of obesity may have yielded conservative estimates of the effect because of the complicated relationship between obesity and physiologic changes that may be associated with these complications. However, when hypertension or diabetes is present in late pregnancy, the decision of obstetrical management including the optimum time and mode of delivery is based on the maternal and fetal wellbeing, regardless of underlying aetiology. Further studies are warranted to refine these relationships.

The findings from the present study reflect pregnancy and labour outcomes for women who received care under a universal publicly funded system where the majority were seen in the first trimester of pregnancy. Women were delivered in academic hospitals with access to highly qualified tertiary care if needed. Although provider preferences may have influenced management of labour and delivery in obese and overweight women, our rates of induction and C-section delivery are within Canadian norms.

This study has several strengths. The characteristics of study sample are reflective of the urban parenting population in Canada, which suggests these results can be generalized. To reduce recall bias associated with the events in labour and at deliver, this data was obtained from medical records. Prospective data collection from detailed questionnaires reduces recall bias and increases accuracy for numerous potential exposures. Finally, this contemporary cohort renders our results highly relevant to current clinical practice.

## Conclusions

In summary, our study demonstrates that among women with term, cephalic, singleton pregnancies who receive prenatal care from a community-based practice, those with pre-pregnancy BMI in the overweight and obese range were at increased risk of pregnancy complications and obstetrical interventions at birth. Obese women with induced labour are at increased risk of C-section. Normal pre-pregnancy BMI is protective for adverse pregnancy and neonatal outcomes and has been associated with less obstetrical interventions.

Increased maternal body weight may be amenable to change prior to pregnancy. Women of reproductive age may benefit from lifestyle counseling to optimize pre-pregnancy weight. Health care professionals should identify increased risk of obesity with every woman of childbearing age in order to address in a timely manner the preventable and modifiable risk factors of obesity. Access of these women to targeted counseling and prevention programs may assist in improving the wellbeing of these women and their families.

## Additional files

Additional file 1: Table S1. Descriptive data for anthropometrics for all study participants. Descriptive data for maternal age, height, pre-pregnancy weight and body mass index (BMI) of the study population presented as mean ± standard deviations (SD) (N = 1996). Additional file 2: Table S2. Adjusted odds ratios for the relationship between maternal obesity prior to pregnancy and mode of delivery stratified by type of labour onset. Multinomial logistic regression models examined the association between maternal pre-pregnancy BMI (normal weight, overweight and obese) and mode of delivery (spontaneous vaginal, emergency caesarean-section), controlling for maternal age, parity, pre-existing health conditions, fertility treatments, history of caesarean section and pregnancy complications, stratified by type of labour onset (spontaneous vs. induced) (N = 1929).

## Abbreviations

BMI: Body mass index
C-section: Caesarean section
OR: Unadjusted odds ratio
aOR: Adjusted odds ratio
CI: Confidence intervals
