# Supplementary material for: Pre-pregnancy Body Mass Index (BMI) and delivery outcomes in a Canadian population
Source: BMC Pregnancy Childbirth. 2014 Dec 20;14:422. doi: 10.1186/s12884-014-0422-y (PMC4300169; doi:10.1186/s12884-014-0422-y)
Supplement: Additional file 1: Table S1. — Descriptive data for anthropometrics for all study participants. Descriptive data for maternal age, height, pre-pregnancy weight and body mass index (BMI) of the study population presented as mean ± standard deviations (SD) (N = 1996). [file 12884_2014_422_MOESM1_ESM.docx]

Additional file 1. Table S1. Descriptive data for anthropometrics for all study participants

| **Characteristics** | Mean ±SD (range) |
| --- | --- |
| Maternal age (years) | 30.6±4.4 (18-43) |
| Maternal height (cm) | 165.9±6.8 (145.0-195.5) |
| Maternal pre-pregnancy weight (kg) | 67.2±13.9 (42.7-150.7) |
| Maternal pre-pregnancy BMI (kg/m^2^) | 24.4±4.8 (18.5-62.8) |

Descriptive data for maternal age, height, pre-pregnancy weight and body mass index (BMI) of the study population presented as mean± standard deviations (SD) (N=1996).
