# Supplementary material for: Pre-pregnancy Body Mass Index (BMI) and delivery outcomes in a Canadian population
Source: BMC Pregnancy Childbirth. 2014 Dec 20;14:422. doi: 10.1186/s12884-014-0422-y (PMC4300169; doi:10.1186/s12884-014-0422-y)
Supplement: Additional file 2: Table S2. — Adjusted odds ratios for the relationship between maternal obesity prior to pregnancy and mode of delivery stratified by type of labour onset. Multinomial logistic regression models examined the association between maternal pre-pregnancy BMI (normal weight, overweight and obese) and mode of delivery (spontaneous vaginal, emergency caesarean-section), controlling for maternal age, parity, pre-existing health conditions, fertility treatments, history of caesarean section and pregnancy complications, stratified by type of labour onset (spontaneous vs. induced) (N = 1929). [file 12884_2014_422_MOESM2_ESM.docx]

**Additional File 2. Table S2. Adjusted odds ratios for the relation between maternal obesity prior to pregnancy and mode of delivery stratified by type of labour onset**

Multinomial logistic regression models examined the association between maternal pre-pregnancy BMI (normal weight, overweight and obese), and mode of delivery (spontaneous vaginal, emergency caesarean section), controlling for maternal age, parity, pre-existing health conditions, fertility treatments, history of caesarean section and pregnancy complications, stratified by type of labour onset (spontaneous vs. induced).

| **Risk Factor** | **MODE OF DELIVERY**^1^ | | | |
| --- | --- | --- | --- | --- |
|  | **Operative vaginal delivery** | | **Caesarean section** | |
|  | ***Induced***  ***labour*** | ***Spontaneous***  ***labour*** | ***Induced***  ***labour*** | ***Spontaneous***  ***labour*** |
| Overweight (BMI 25-29.9kg/m^2^)^2^ | 1.5; 0.8-2.7 | 1.1; 0.7-1.8 | 1.1; 0.7-2.0 | 1.1; 0.6-1.8 |
| Obesity (BMI ≥ 30 kg/m^2^)^2^ | 0.4; 0.1-1.4 | 0.3; 0.1-1.0 | **2.2; 1.2-4.1**** | 1.5; 0.7-3.0 |
| Maternal age (>35 years old)^3^ | 1.1; 0.6-2.3 | 1.3; 0.8-2.1 | 1.4; 0.8-2.5 | **1.7; 1.0-2.7**** |
| Parity (multipara)^4^ | **0.2; 0.1-0.5*** | **0.1; 0.1-0.2*** | **0.08; 0.03-0.1*** | **0.8; 0.04-0.1*** |
| Pre-existent health conditions^5^ | 1.4; 0.6-3.2 | 1.0; 0.5-2.0 | 1.5; 0.8-2.8 | 0.8; 0.4-1.5 |
| Fertility treatments^6^ | 0.6; 0.2-1.7 | 1.0; 0.4-2.5 | 0.5; 0.2-1.2 | 1.8; 0.4-3.8 |
| Previous caesarean section^7^ | 1.7; 0.2-14.9 | **2.9; 1.1-7.5**** | **33.3; 10.7-104.0*** | **24.2; 11.2-52.5*** |
| Pregnancy complications^8^ | 1.1; 0.6-1.9 | 1.2; 0.6-2.5 | 0.8; 0.5-1.3 | 1.2; 0.6-2.4 |

Data presented as adjusted odds ratio; 95% confidence intervals

^1^Reference category: spontaneous vaginal delivery

^2^Reference category: normal weight (BMI 18.5-24.9kg/m^2^)

^3^Reference category: maternal age at delivery less than 35 years old

^4^Reference category: nullipara

^5^Pre-existent maternal health conditions include diabetes mellitus, hypertension, chronic heart and renal diseases. Reference category: no previous health conditions

^6^Fertility treatments include the use of fertility enhancing drugs, artificial insemination and artificial reproductive techniques such as *in vitro* fertilization, intracytoplasmic sperm injection, fresh and donor embrio transfer etc. Reference category: no fertility treatments (spontaneous conception)

^7^Reference category: no previous caesarean section delivery

^8^Pregnancy complications include gestational diabetes, preeclampsia, eclampsia, placental abruption, placenta praevia. Reference category: no pregnancy complications

*****p< 0.001; ******p<0.05
